# List of Reviewers

**DOI:** 10.1017/jns.2023.6

**Published:** 2023-04-27

The Editorial Board of *Journal of Nutritional Science* would like to thank the following for their contribution as peer reviewers in 2022:

- Mateen Abbas
- Yasir Abdullahi
- Joana Abou-Rizk
- Katherine Adams
- Emre Adigüzel
- Tariku Afata
- Pooyan Afzali Harsini
- Sixtus Aguree
- Lyutha Al Subhi
- Mohammed Al-Beltagi
- Ute Alexy
- Cláudia Almeida
- Charles Apprey
- Joshua Arimi
- Omar Elind Arroyo-Helguera
- Elaheh Asgari
- Junaida Astina
- Fei Au-Yeung
- Riddhi Babel
- Mahnaz Bahri Khomami
- Diane Bairamian
- Nazal Bardak Perçinci
- Ziad Bassil
- Paula Bautista-Niño
- Maria Angela Bellomo-Brandao
- Brabon Benard
- Annemarie Bennett
- Anteneh Berhane
- Jennifer Bernal Rivas
- Hanne Christine Bertram
- Julia Bird
- Christopher Birt
- Zebenay Workneh Bitew
- Anne Bjerregaard
- Maria Luisa Bonet Piña
- Anne-Marie Bostrom
- Paul Breslin
- Andrea Bulungu
- Raquel Canuto
- Ligia Carlos
- Sharayah Carter
- Ronna Chan
- Whye Lian Cheah
- Guoxun Chen
- Justin Chileshe
- Tarun Choudhary
- Maria Choukri
- Jean-Pierre Chouraqui
- Robin Christensen
- Zachary Clayton
- Marie Conway
- Jennifer Crowley
- Shaonong Dang
- Thales da Silva
- Melissa de Araújo
- Monica de Assunção
- Bethelhem Debela
- Andrea Deierlein
- Cristian Del Bo
- Muzaffer Denli
- Aly Diana
- Tona Diddana
- Mariana Dineva
- Valeri Dipasquale
- Haibo Dong
- Pamela Dyson
- Melaku Engidaw
- Tommaso Ercoli
- Christopher Esezobor
- Elizabeth Eveleigh
- Waseem Fatima
- Awat Feizi
- Fentaw Feleke
- Elisa Felix-Soriano
- Artur Ferron
- Maryah Stella Fram
- David Fraser
- Aya Fujiwara
- Teressa Fung
- Sanjeev Galande
- Christopher Gardner
- Malin Garemo
- Heath Gasier
- Tafere Gebreegziabher
- Dereje Getahun
- Eileen Gibney
- Birtucan Gizachew
- Andrea Glenn
- Zeynep Goktas
- Maria Gombi-Vaca
- Filomena Gomes
- Cristiana Gontijo
- Danielle Gonçalves
- Xiong Guo
- Gloria Yareli Gutierrez-Silerio
- Mihnea-Alexandru Găman
- Ella Haddad
- Sumanto Haldar
- Ruhaya Hasan
- John Hayes
- Jessica Heckert
- Harri Hemilä
- Oscar Herran
- Yasuki Higashimura
- Daniel Hinojosa-Nogueira
- Michael Holick
- Graham Horgan
- Gideon Iheme
- Francois Iris
- Urszula T. Iwaniec
- Carol Johnston
- Vanessa Jugha
- Marzieh Kafeshani
- Michael Kale
- Alexandra Karachaliou
- Nena Karavasiloglou
- Spyridon Karras
- Berhanu Kibemo
- Mary Koenig
- Aruna Jyothi Kora
- Scott L. Kronberg
- Slawomir Jozef Krzebietke
- Solo Kuvibidila
- Elisabeth Kylberg
- Marjaana Lahti-Koski
- Abera Lambebo
- Anna Lartey
- Avula Laxmaiah
- Matthew Lee
- Zhansheng Li
- Kuang Hock Lim
- Changqi Liu
- Jufen Liu
- Junhua Liu
- Caitlin M. Lowery
- Gary Ma
- Fernanda Madruga
- Hayato Maeda
- Eugenia Manukhina
- Tonderayi Matsungo
- Nirupa Matthan
- Heather Mechler
- Nana Mikami
- Lorenza Mistura
- Sanjay K. Mohanty
- Juan Moreno Aznarez
- Grace Motey
- Vishal Mudgal
- Karen Mumme
- Courtney Neal
- Dwi Adi Nugroho
- Dominika Ochnik
- Riceli Oliveira
- Audrey Opoku-Acheampong
- Vahidreza Ostadmohammadi
- Sherly M. Parackal
- Indira Paz-Graniel
- Sally Poppitt
- Harsh Priya
- Frederick D. Provenza
- Edward Quadros
- Ana Renteria-Mexia
- Lucas Ribeiro
- Nikolaos Rodopaios
- Cíntia Rodrigues
- Nancy Rodriguez
- Imogen Rogers
- Mahama Saaka
- Ferguson Saapiire
- Katharina Anne Scherf
- Karen Scott
- Cecilia Severi
- Qun Shen
- Arif Jamal Siddiqui
- Anne Sidnell
- K D Renuka Ruchira Silva
- Tippawan Siritientong
- Juliana Souza
- Regina Spadari-Bratfisch
- Nikoleta Stamataki
- Tonje Stea
- Mariano Stornaiuolo
- Erliera Sufarnap
- Chiara Tabasso
- Degnet Teferi Asres
- Wondewosen Teklesilase
- Judith ten Have
- Yalcin Tepe
- Esubalew Tesfahun
- Barbara Thomson
- Chalachew Tiruneh
- Moesis Tolentino
- Analía Lorena Tomat
- Linde van Lee
- Divya Vanoh
- Aswathy Vijayakumar
- Katia Wagner
- Kai Wang
- Leigh Ward
- Janet Weber
- Camila Weschenfelder
- R. Douglas Wilson
- Zelalem Wondimagegne
- Yusni Yusni
- Ayah Zaidalkilani

